# Exposure to arsenic and other potentially toxic elements: health risk assessment and source analysis in the Wuming Basin, Guangxi Province, China

**DOI:** 10.1038/s41598-024-52947-y

**Published:** 2024-02-03

**Authors:** Bo Hu, Jie Li, Rui Liu, Guoxin Lei, Xinyu Wang, Lei Wang

**Affiliations:** 1 Key Laboratory of Environment Change and Resources Use in Beibu Gulf, Ministry of Education, Nanning Normal University, 175 Mingxiu East St., Nanning, 530001 People’s Republic of China; 2 School of Environmental and Life Sciences, Nanning Normal University, Nanning, 530001 People’s Republic of China; 3 Geological Survey of Guangxi Zhuang Autonomous Region, Nanning, 530023 People’s Republic of China; 4 College of Resources and Environment, Yangtze University, Wuhan, 430100 People’s Republic of China

**Keywords:** Environmental sciences, Solid Earth sciences, Risk factors

## Abstract

Guangxi, China, is one of the world's largest karst regions where potential toxic elements tend to accumulate, resulting in high soil background values. This study explores the ecological risk, elemental baseline values, and sources of potential toxic elements in karst regions, expanding the research to include 21 common elements. The significance of this research lies in its implications for the management of potential toxic element pollution, the formulation of environmental quality standards, and soil remediation in karst areas. In this study, 12,547 topsoil samples (0–20 cm) were collected in the study area. Pollution assessment and ecological risk evaluation of eight potential toxic elements (Zn, Ni, Cu, Pb, Cd, Hg, Cr, and As) were conducted using the geo-accumulation index method and potential ecological risk index method. Multivariate statistical analysis was applied to analyze the total content of 21 common elements (Zn, Ni, Cu, Pb, P, Cd, Hg, Co, Mn, Cr, V, I, S, As, pH, Se, N, CaO, Corg, Mo, and F). Additionally, the potential sources of 21 soil elements were preliminarily quantitatively analyzed using the principal component analysis-absolute principal component scores-multiple linear regression receptor model. The results showed that (1) Zn, Ni, Cu, Pb, Cd, Cr, V, and As were enriched in the research area and Ca, Cd, Mn, Mo, Hg, As, and Cu might have been influenced by human activities; (2) Cr, Pb, As, and Zn were generally lightly polluted, with Hg having a moderate potential ecological risk level; and (3) Ni and Zn have contributions of 37.99% and 35.07% from geological sources, agricultural fertilization, and pesticides. Mo, V, Cr, Se, Hg, and As exhibit contributions ranging from 39.44 to 59.22% originating from geological backgrounds and human activities. Corg, S, N, and P show contributions of 45.39% to 80.33% from surface vegetation. F, Co, Mn, and Pb have contributions ranging from 31.63 to 47.93% from acidic rocks in the soil parent material, mining activities, and transportation. Cd and CaO derive 31.67% and 40.23%, respectively, from soil parent material and industrial sources. I has 31.94% from geological background and human activities, and 31.95% from soil parent material and atmospheric sources. Cu has 30.56% from geological sources. The study results can serve as a scientific basis for element research in karst areas domestically and internationally.

## Introduction

Potential toxic elements (PTEs) are commonly present in the soil environment, characterized by traits such as being difficult to degrade, causing persistent harm, irreversible effects, and accumulation in the food chain^1,2^. They have various negative impacts on human health. Arsenic (As) can cause nausea, vomiting, a reduction in the production of red and white blood cells, abdominal pain, and other adverse health effects^3^. Hexavalent chromium (Cr VI) can stimulate nasal mucosa, leading to a runny nose, nasal ulcers, cough, asthma, and respiratory difficulties. Prolonged exposure to this element can also result in liver damage, kidney failure, circulatory issues, and neurological disorders^4,5^. Cadmium (Cd), chromium (Cr), copper (Cu), lead (Pb), and zinc (Zn) can migrate in different environments, being absorbed by the human body and posing a potential threat to human health^6^. Among them, Cd and Pb are highly toxic elements that, even at very low levels, can cause kidney damage and may lead to reduced reproductive ability, liver dysfunction, high blood pressure, and tumors^3,7^.

PTEs in soil have both natural and anthropogenic sources. Natural sources primarily include rock weathering, soil erosion, and volcanic eruptions, with exposed rocks being the predominant source of soil material^8,9^. The rock type of the parent material determines the degree of weathering, thereby controlling the geochemical behavior of elements during soil formation and influencing the extent of element enrichment in the soil^10,11^. Generally, PTEs tend to accumulate in alkaline soils^12^. Anthropogenic activities mainly involve industrial processes and agricultural practices. Industrial activities such as mining, smelting, electroplating, solid waste disposal, and factory emissions, as well as agricultural practices such as sewage irrigation and the use of fertilizers, contribute to the introduction of PTEs into the soil^13,14^.

At present, research on the pollution assessment and sources of PTEs tends to focus on areas with higher human activity intensity^15,16^, paying attention to the impact of industrial and agricultural development on PTEs^17^. However, studies on PTEs in areas with high geological backgrounds are gradually being conducted. China has the world's largest karst area^18^, and Guangxi, as one of the largest karst provinces in China, has widespread distribution of carbonate rocks. The karst topography in karst regions leads to the leaching of parent rocks, enrichment of PTEs, and elevated background levels of PTEs in the soil^19^. However, there are few studies on the baseline values and enrichment status of PTEs based on different geological units, soil types, and land-use practices. Additionally, there is limited research on pollution assessment and source analysis, often focusing on a few common PTEs. Examples include pollution assessment and source analysis of seven PTEs (Cd, As, Pb, Cr, Cu, Ni, Zn) in the karst region of northwest Guizhou^20^, source analysis of six heavy metals (Cd, Cr, Cu, Ni, Pb, Zn) in agricultural soils in Wenzhou, Zhejiang^21^, as well as pollution characteristics and source analysis of individual heavy metals such as Cd^16,22^, and Mo^23^. In contrast, this study uses the example of the Wuming Basin in Guangxi, expanding the research scope to 21 elements (CaO, Corg, pH, As, Cd, Co, Cr, Cu, F, Hg, I, Mn, Mo, N, Ni, P, Pb, S, Se, V, Zn). It thoroughly discusses the baseline values, enrichment status, and sources of these elements in karst areas with high geological backgrounds, providing a scientific basis for the formulation of environmental quality standards for soil in this region and other karst areas. This study holds significant importance for soil element research and soil restoration management in karst areas.

To ensure the accuracy of research results, this study collected tens of thousands of samples in a research area of 1380 km^2^, focusing on the study of 21 common elements. The objectives of this study are: (1) to propose soil geochemical baseline values for the 21 elements in the study area based on different geological units, soil types, and land-use practices; (2) to conduct pollution assessment in the study area using the geo-accumulation index (${I}_{{\text{geo}}}$) and potential ecological risk index (PERI); (3) to quantitatively analyze the potential sources of soil elements using the principal component analysis-absolute principal component scores-multiple linear regression (PCA-APCS-MLR) receptor model, in conjunction with geological analysis, soil analysis, and land-use type analysis".

## Materials and methods

### Description of the research zone

**Figure 1:**
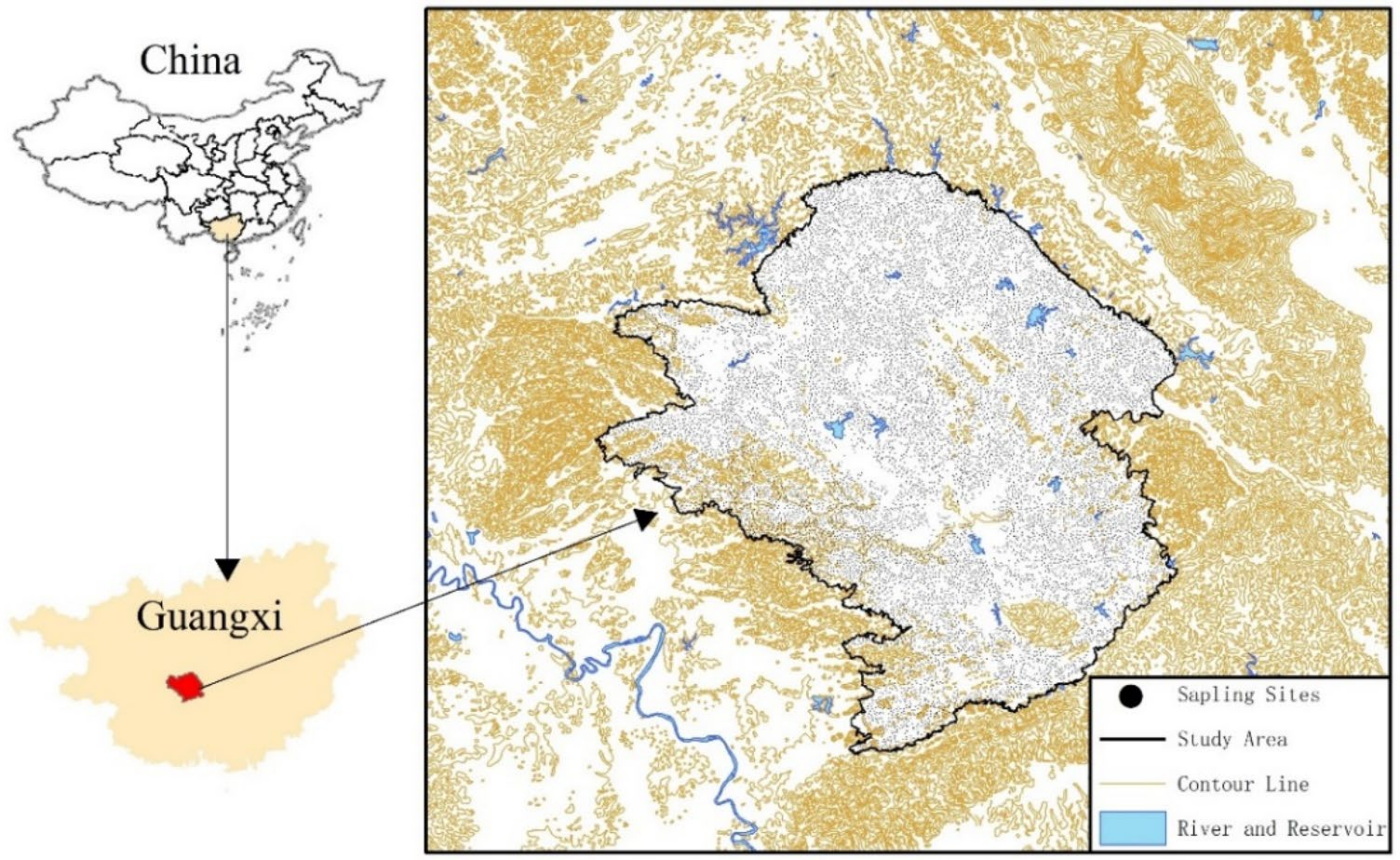
Map of sampling locations. Software: ArcGIS 10.8. https://www.esrichina.hk/en-hk/home (ESRI, US).

**Figure 2:**
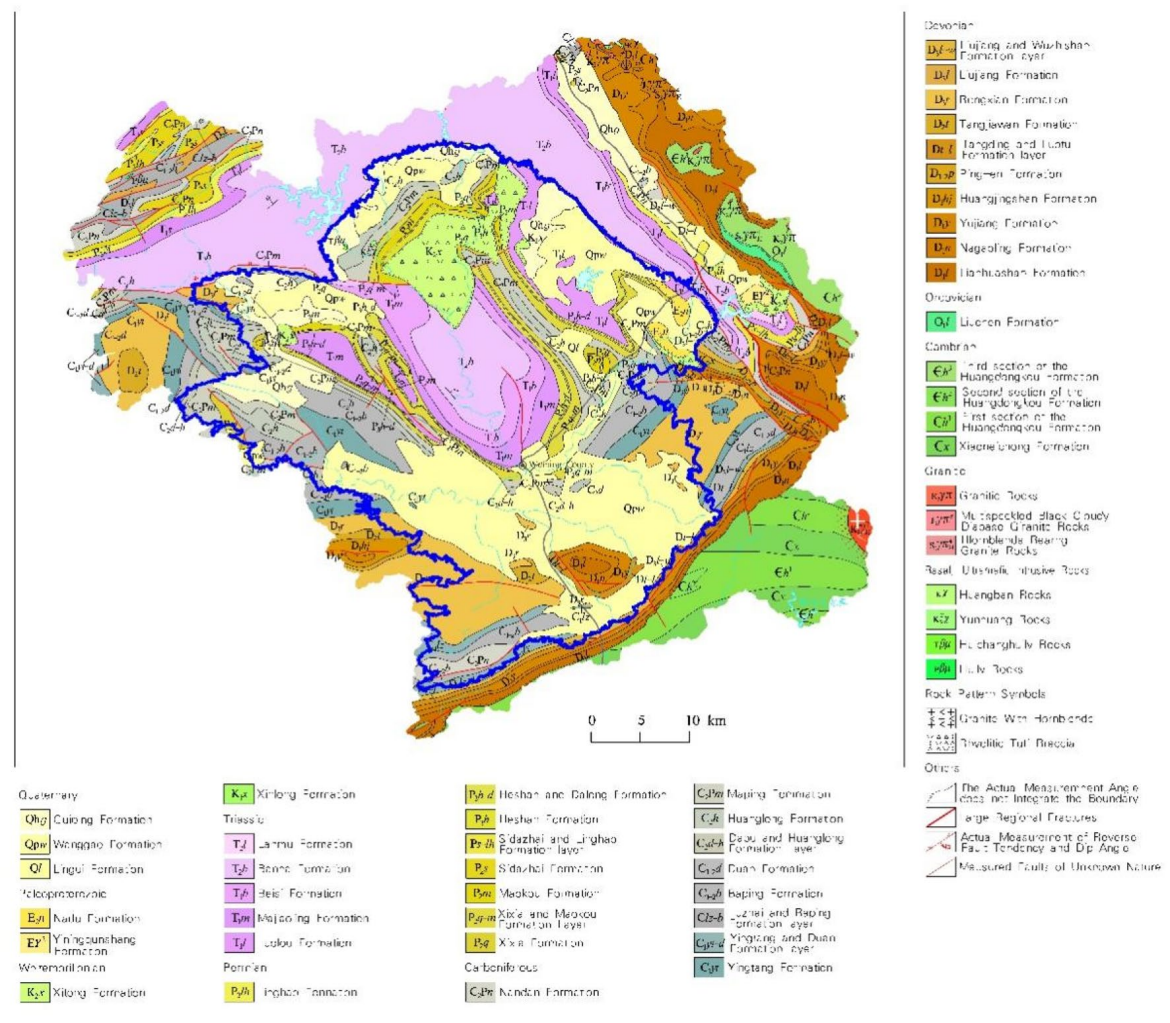
Geology map of the research zone. Software: MapGIS 6.7. https://www.mapgis.com/ (Wuhan Zhongtian Network Co., Ltd).

The research region is situated in the central basin of Wuming District in the middle part of southern Guangxi, China (107°55′–108°27′ E, 22°51′–23°27′ N) and occupy a joint area of 1380 km^2^ (Fig. 1). It has a flat terrain with thick soil layers. The largest areas are covered by paddy fields and drylands, which are contiguous. This region is part of the southern subtropical monsoon climate zone, with abundant light, heat, and rainfall. Within the study area, Devonian, Carboniferous, Permian, Triassic, Cretaceous, Palaeogene, and Quaternary sedimentary strata are exposed (Fig. 2). The parent materials for soil formation mainly include quaternary sediments and clastic, carbonate, neutral, and acidic rocks. Brick-red ferruginous soil is the most widely distributed soil, followed by rice soil, which together account for 87% of the evaluation range area (Fig. S1). Various minerals have been discovered. The main land use types are paddy fields and drylands, and a widespread distribution of forests, orchards, tea gardens, factories, roads, and urban buildings (Fig. S2). The total area of the Wuming Karst basin is 4536.3 km^2^, with the karst area covering approximately 3175.7 km^2^. It is characterized by various landforms, including peak-clustered basins, valleys, peak-forested valleys, isolated peak-hilly valleys, and karst plains. Geological structures developed mainly in the northwest and northeast directions. Karst development and karst groundwater are mainly moderately abundant, followed by abundant and scarce karsts^24^.

### Sample collection and analysis

The specimens were gathered and arranged in water paddy fields and dryland plots at an average sampling density of 8–12 samples per km^2^. Global Positioning System coordinates were used as reference points and 4–6 subsampling points were determined within a range of 50–100 m from each reference point. The sub-samples were merged to form a combined specimen. When the sampling area was rectangular, an 'S' pattern was used to arrange the subsampling points. When the sampling area was approximately square, an 'X' or checkerboard pattern was used. Continuous soil columns from 0 to 20 cm were collected and the roots, straw, stones, and organisms were manually removed. The subsampling points were thoroughly mixed, resulting in 12,547 agricultural soil specimens. Subsequently, pre-processing was conducted by air-drying the soil samples in the laboratory, followed by crushing and sieving through a 2-mm pore-size filter. The specimens were tested using various methods, including inductively coupled plasma mass spectrometry, inductively coupled plasma optical emission spectrometry, X-ray fluorescence spectrometry, the volumetric method, and the glass electrode method (pH). Elements (Mo, Cd, Ni, Co, B, I, Mn, Cu, V, Pb, Zn, Cr, P, S, As, Hg, Se, F, and N), major components (CaO), organic matter content, and soil pH were analyzed. For the whole analysis process, the national soil primary standard material (GSS-1) was used as the quality control standard. The recovery rate of all elements was within the range of 100 ± 10%.

### Methods for PTEs pollution assessment

An assessment of topsoil PTEs was conducted on the degree of enrichment and contamination of agricultural land layers in the Wuming Basin using the ${I}_{{\text{geo}}}$ and potential ecological risk index (PERI) methods. The ${I}_{{\text{geo}}}$ method considers diagenesis and provides a relatively accurate assessment of natural or anthropogenic pollution^25–27^, while the PERI approach focuses on estimating the ecological hazards of PTEs.

${I}_{{\text{geo}}}$ was calculated using the following equation ^28^:

$${I}_{{\text{geo}}}={log}_{2}\frac{{C}_{n}}{1.5{B}_{n}}$$

where ${C}_{n}$ is the measured content (mg kg^−1^) of element n in the soil, ${B}_{n}$ is the corresponding background value (mg kg^−1^) for the element, and 1.5 is the constant for the natural fluctuation of the PTE element content during diagenesis. ${I}_{{\text{geo}}}$ can be applied to assess the level of soil contamination (Table S1). This method considers the contribution of background values due to naturally occurring geological processes and the impact of anthropogenic contributions to PTE contamination.

The PERI was calculated as follows^29^:

$${C}_{f}^{i}=\frac{{C}_{i}}{{C}_{n}^{i}}; {E}_{r}^{i}={T}_{r}^{i}\times {C}_{f}^{i}; RI={\sum }_{i=1}^{n}{E}_{r}^{i}$$

where ${C}_{f}^{i}$ is the pollution exponent of a specific PTE; ${C}_{i}$ and ${C}_{n}^{i}$ are the measured values of PTE concentration and the reference value or background value for a certain HM; ${T}_{r}^{i}$ is the toxic response coefficient for a specific PTE (the toxic response coefficients for Hg, Cd, As, Pb, Cu, Ni, Cr, and Zn are 40, 30, 10, 5, 5, 5, 2, and 1, respectively)^29^; ${E}_{r}^{i}$ is the PERI for an individual PTE; and $RI$ is the PERI for several PTEs. The classification criteria for the potential ecological risk levels are listed in Table S1.

### Methods for source analysis of PTEs

To identify the origins of PTEs, the coefficient of variation (CV), correlation analysis, principal component analysis (PCA), and PCA-Absolute Principal Component Scores-Multiple Linear Regression (APCS-MLR) model were used to analyze the sources of PTEs in the topsoil of the Wuming Basin in Guangxi. The CV, correlation, and PCA analysis can qualify the origins of contamination. A strong correlation among elements belonging to the same principal component suggests that the sources may be similar or the same. Based on PCA, quantitative analysis of the origins of PTEs can be conducted utilizing the normalized APCS of the normalized PTE content, as well as the multivariate linear regression receptor model (MLR). This approach helps to determine the contribution of each principal component to PTE contamination and assesses the extent of the contribution of these principal components to different PTEs.

The following are the primary procedures of PCA-APCS-MLR^30^:

1. After standardizing the data, a zero concentration factor was introduced.
  $${({Z}_{0})}_{i}=\frac{0-{\overline{C}}_{i}}{{\sigma }_{i}}$$
2. The APCS was obtained by subtracting ${Z}_{0}$ from the PCA-normalized factor scores.
3. Multiple linear regression was performed with APCS as the independent variable and PTE concentration as the dependent variable, and the regression coefficients were utilized to compute the origin contribution of each PTE:
  $${C}_{i}={b}_{i0}+{\sum }_{p=1}^{p}\left({b}_{pi}\times {APCS}_{p}\right)$$
  where ${\overline{C}}_{i}$ is the mathematical mean of the PTE content, ${\sigma }_{i}$ is its standard deviation, ${C}_{i}$ is the content estimate, ${b}_{i0}$ is the constant term of the multivariate linearity, ${b}_{pi}$ is the regression coefficient of the multivariate linearity, and ${b}_{pi}\times {APCS}_{p}$ is the mean source p-contribution value.

## Results and discussion

### Descriptive statistics

The descriptive statistical results for 19 common soil elements, pH, and Corg are presented in Table S2. The pH of the soil ranges from 3.22 to 8.42, with an average of 5.29. The content of soil Corg varies from 2.00 to 60.80 mg/kg, with an average concentration of 13.00 mg/kg. The average values for Zn, Ni, Cu, Pb, P, Cd, Co, Mn, Cr, V, I, As, Se, CaO, Mo, F, and Hg are 132.07, 37.94, 34.02, 40.98, 926.42, 0.52, 11.47, 552.57, 182.67, 176.66, 7.66, 39.62, 0.85, 0.50, 2.21, 506.88 mg/kg, and 181.98 μg/kg, respectively.

From Table S2, it can be observed that compared to the national soil (Layer A) ^31^, the study area exhibits a high abundance of Zn, Ni, Cu, Pb, Cd, Hg, Cr, V, I, As, and Se but a lower CaO content. The Co, Mn, Mo, and F contents were similar. In comparison with Guangxi soil (Layer A) ^32,33^, the contents of Zn, Ni, Cu, Pb, Cd, Hg, Mn, Cr, V, As, and CaO were higher, the Mo content was lower, and the Co, I, Se, and F contents were similar. Compared with the surface soil in the Guangxi Beibu Gulf Economic Zone^34^, the contents of Zn, Ni, Cu, Pb, P, Cd, Hg, Co, Mn, Cr, V, I, As, Se, CaO, Mo, and F were higher but the N and Corg contents were lower. Among them, 95.28%, 83.47%, and 98.30% of the samples exceed the soil background values of China, Guangxi, and the Northern Gulf Economic Zone of Guangxi for chromium (Cr), respectively. Zn, Ni, Cu, Pb, Cd, Cr, V, and As were enriched in the Guangxi Wuming Basin, showing significant differences from the national, Guangxi, and Guangxi Beibu Gulf Economic Zone soil background values.

The coefficient of variation (CV) reflects the uniformity of sample data and can be used to indicate the degree of influence of human activities on various elements^35,36^. The CV of soil elements in the research zone, from largest to smallest, are CaO > Cd > Mn > Mo > Hg > As > Co > Zn > Cr > I > Ni > Cu > Pb > P > V > F > Se > N > S > Corg > pH. A CV ≤ 0.2 indicates low variability; 0.2 < CV ≤ 0.5 indicates moderate variability; 0.5 < CV ≤ 1 indicates high variability; and CV > 1 indicates extremely high variability^37^. In the study area, only pH had a CV less than 0.2, indicating little difference in soil acidity and alkalinity across the region. The Corg, S, N, Se, F, V, and P levels exhibited moderate variability. Mo, Hg, As, Co, Zn, Cr, I, Ni, Cu, and Pb exhibited high variabilities. CaO, Cd, and Mn displayed extremely high variabilities, and the coefficients of variation are 3.47, 1.71, and 1.41, respectively (Table S2). The preliminary analysis suggested that 13 elements may be influenced by point-source pollution: CaO, Cd, Mn, Mo, Hg, As, Co, Zn, Cr, I, Ni, Cu, and Pb. Among these 13, CaO, Cd, Mn, Mo, Hg, As, and Cu had relatively large skewness, kurtosis, and CV, further indicating strong spatial heterogeneity for these elements, likely influenced mainly by anthropogenic factors.

### Soil geochemical baseline value

Based on the different parent rock lithologies in the study area, 12,547 surface soil samples were divided into five statistical units: quaternary sediments, acidic rocks, clastic rocks, carbonate rocks, and neutral salts^17,38^. The statistical results of the geochemical baseline values for surface soils are presented in Table S3. According to different soil types, the samples were divided into five statistical units: red soil, lime soil, paddy soil, alluvial soil, and purple soil. The statistical results of the geochemical baseline values for agricultural soils of different soil types are shown in Table S4. Based on different land-use practices, the samples were divided into two statistical units: dryland and paddy fields. The statistical results of the geochemical baseline values for agricultural soils under different land-use practices are provided in Table S5. EF = background value of the statistical unit/background value of the study region.

According to the geochemical baseline values for agricultural soils in different geological units provided in Table S3, it can be observed that in acidic rock soils, the average contents of Mn, As, Cd, Pb, Co, CaO, F, N, and Corg are all higher than in other soils, with values that are 4.2 times, 3.1 times, 2.8 times, 2.5 times, 2.5 times, 2.1 times, 2.0 times, 1.4 times, and 1.3 times higher than the lowest contents in other soils, respectively. Cu has the highest content in clastic rock soils. In carbonate rock soils, the contents of Cr, Hg, I, Mo, Ni, P, S, Se, V, and Zn are 200.85, 173, 9.78, 2.36, 37.1, 895, 277.1, 0.913, 203, and 120.1 ug/g, respectively, which are higher than in the other four parent rock soils. The enrichment coefficients follow the same trend as the contents. In acidic rock soil samples, the contents of As and Cd are 49.5 mg/kg and 0.318 mg/kg, respectively, exceeding the risk screening values^39^, and they have the highest exceedance rate among the five parent rock soils. In carbonate rock soil samples, the contents of As and Cr exceed the standards by 14.3% and 33.9%, respectively.

According to the geochemical baseline values for agricultural soils in different soil types presented in Table S4, it can be observed that in lime soil, the average contents of Cd, Mn, As, Zn, Mo, Ni, Cr, Co, Pb, V, Cu, P, CaO, S, N, and Corg are all higher than in other soils, being 5.2, 4.2, 3.8, 3.7, 3.5, 3.2, 2.7, 2.6, 2.5, 2.2, 2.1, 1.9, 1.6, 1.5, 1.4, and 1.3 times higher than the lowest contents in other soils, respectively. Hg, I, and Se have the highest contents in red soil, while P has the highest content in purple soil. Se is enriched in red soil, and F is enriched in purple soil. The content of Cr exceeds the standard in 7.9% of red soil samples, and in lime soil samples, the contents of As, Cd, Cr, and Zn are 50.8, 0.794, 243.1, and 265.95 mg/kg, respectively, exceeding the risk screening values. Lime soil has the highest exceedance rate among the five soil types. The results indicate that Cr is the main potentially toxic element (PTE) pollutant in the Wuming Basin, and lime soil is the most severely affected by PTE pollution.

According to the geochemical baseline values for agricultural soils under different land use types presented in Table S5, it can be observed that in dryland, the contents of I, Mo, As, and Cr are much higher than in paddy fields, being 5.8, 3.2, 2.9, and 2.1 times higher than the contents in paddy fields, respectively. As, Co, Cu, I, Mn, Mo, Ni, Pb, Se, V, and Zn are enriched in dryland, while Corg, F, N, and S are enriched in paddy fields. The content of Cr exceeds the standard in 16.3% of the dryland soil samples.

### Evaluation of PTE contamination

#### Geoaccumulation index (${I}_{{\text{geo}}}$)

**Figure 3:**
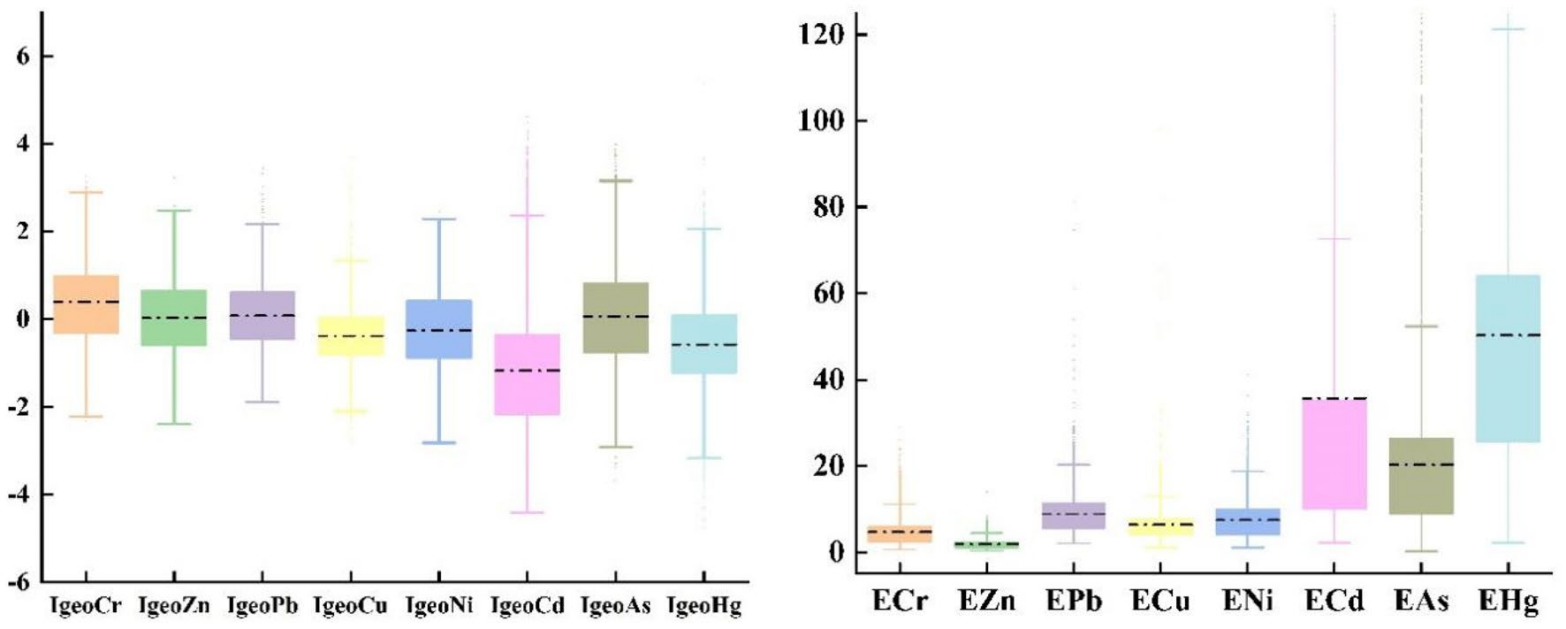
Box-line diagram of soil PTEs ${I}_{{\text{geo}}}$ and PERI. The Box-line Diagram show the minimum value (lower whisker), 25th quartile, median, 75th quartile, and maximum value (upper whisker) and outlier (dot).

**Figure 4:**
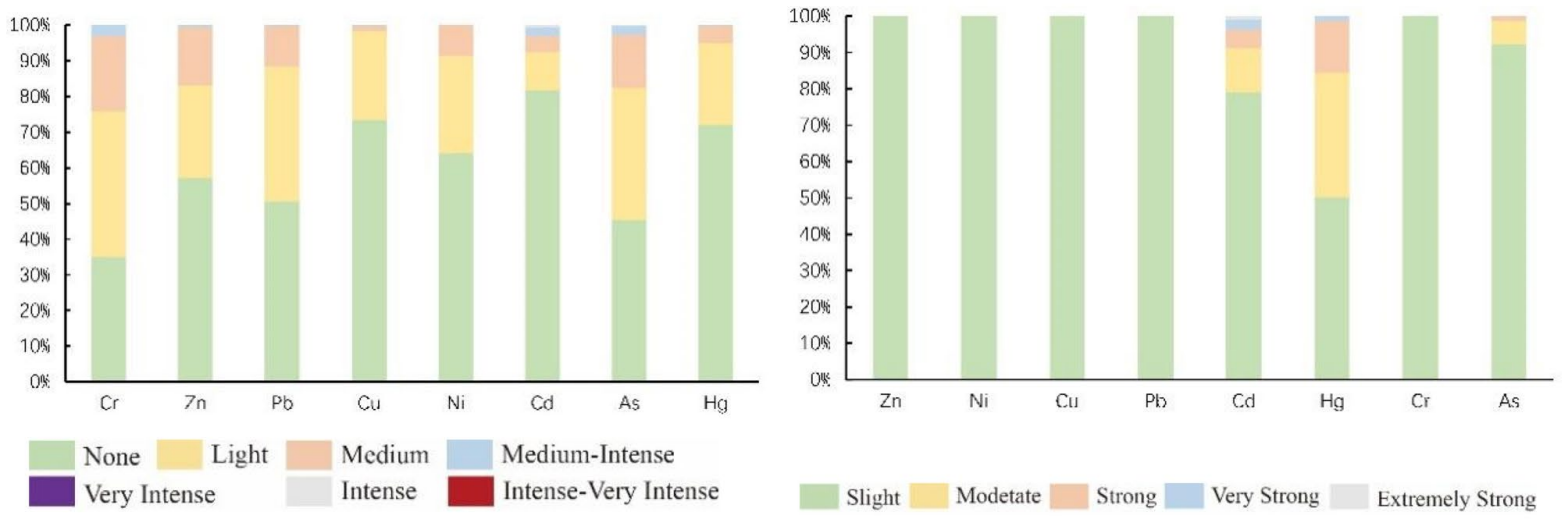
Frequency distribution histogram of soil heavy metal ${I}_{{\text{geo}}}$ and PERI.

In order to further assess the pollution levels of the elements, the ${I}_{{\text{geo}}}$ was calculated as a reference^35^. The analysis results of ${I}_{{\text{geo}}}$ are shown in Fig. 3, ranging from −6.20 to 5.37. The mean ${I}_{{\text{geo}}}$ for various PTEs in descending order was Cr (0.39) > Pb (0.07) > As (0.06) > Zn (0.02) > Ni (−0.25) > Cu (−0.38) > Hg (−0.58) > Cd (−1.17). The ${I}_{{\text{geo}}}$ averages of Cr, Pb, As, and Zn were greater than 0, indicating slight pollution, whereas the remaining elements had averages below 0, indicating no pollution. Figure 4 shows the distribution of sample points corresponding to different land accumulation index classes in the research region. The sample points for Cr, Pb, As, and Zn were distributed among five pollution levels: none, light, medium, medium intensity, and intense. Among these, Cr showed the most severe pollution, with only 35.20% of the sample points categorized as no pollution, 40.69% as slight pollution, 21.05% as moderate pollution, 3.04% as moderate-to-strong pollution, and 0.02% as strong pollution. Although Hg and Cd were generally in unpolluted states (average value < 0), some individual sample points had high pollution levels. For Hg, 71.91%, 22.84%, 4.94%, 0.29%, 0.02%, and 0.01% of the sample points were categorized as none, light, moderate, medium, medium intensity, intense, and very intense, respectively. For Cd, 81.80%, 10.50%, 4.61%, 2.35%, 0.68%, and 0.06% of the sample points were categorized as none, light, moderate, medium, medium-intensity, intense, and strong, respectively. In addition, 3.06% of the sample sites were medium-intensity contaminated and above for Cr and 3.03% of the sample sites were medium-intensity contaminated and above for Cd. Overall, the comprehensive analysis indicated that the PTEs Cr, Pb, As, and Zn in the research region were generally lightly polluted, with localized soil contamination from Cd.

### Potential ecological risk index

The average PERI for the individual elements was ranked as follows: Hg (50.34), Cd (35.69), As (20.26), Pb (8.85), Ni (7.460), Cu (6.40), Cr (4.68), and Zn (1.84) (Fig. 3). Both Hg and Cd exhibited sample distributions across different risk levels. As shown in Fig. 4, 50.16% of the samples had slight ecological risk, 34.10% had moderate ecological risk, 14.30% had strong ecological risk, 1.35% had very strong ecological risk, and 0.08% had extremely high ecological risk. For Cd, 78.95% of the samples had slight ecological risk, 12.21% had moderate ecological risk, 5.14% had strong ecological risk, 2.71% had very strong ecological risk, and 0.99% had extremely strong ecological risk. Among the samples, 92.22% were in the slight ecological risk category, 6.44% in the moderate ecological risk category, 1.25% in the strong ecological risk category, and 0.09% in the very strong ecological risk category. No samples were in the extremely strong ecological risk category. Ni, Cu, and Zn accounted for > 99.90% of the samples in the slight ecological risk category, whereas the others were distributed within the moderate ecological risk level. The ecological risk levels for Zn and Cr were in the slight category, with PERI values below 40. The comprehensive PERI has an average value of 135.52, ranging from 18.58 to 2602.42, indicating a generally slight to moderate ecological risk. The results showed that 69.78% of the samples have a risk index (RI) < 150, indicating a slight ecological risk; 24.11% have 150 ≤ RI < 300, indicating a moderate ecological risk level; 5.55% have 300 ≤ RI < 600, indicating a strong ecological risk; 0.57% have RI ≥ 600, demonstrating a very strong ecological risk. Considering the comprehensive ${I}_{{\text{geo}}}$ results, although Hg is not overall contaminating, its underlying ecological risk level is moderate because of its significant toxicity. Although the overall ecological risk level was slight to moderate, some localized areas had relatively high ecological risk levels.

### Analysis of elements sources

#### Relevance analysis

**Figure 5:**
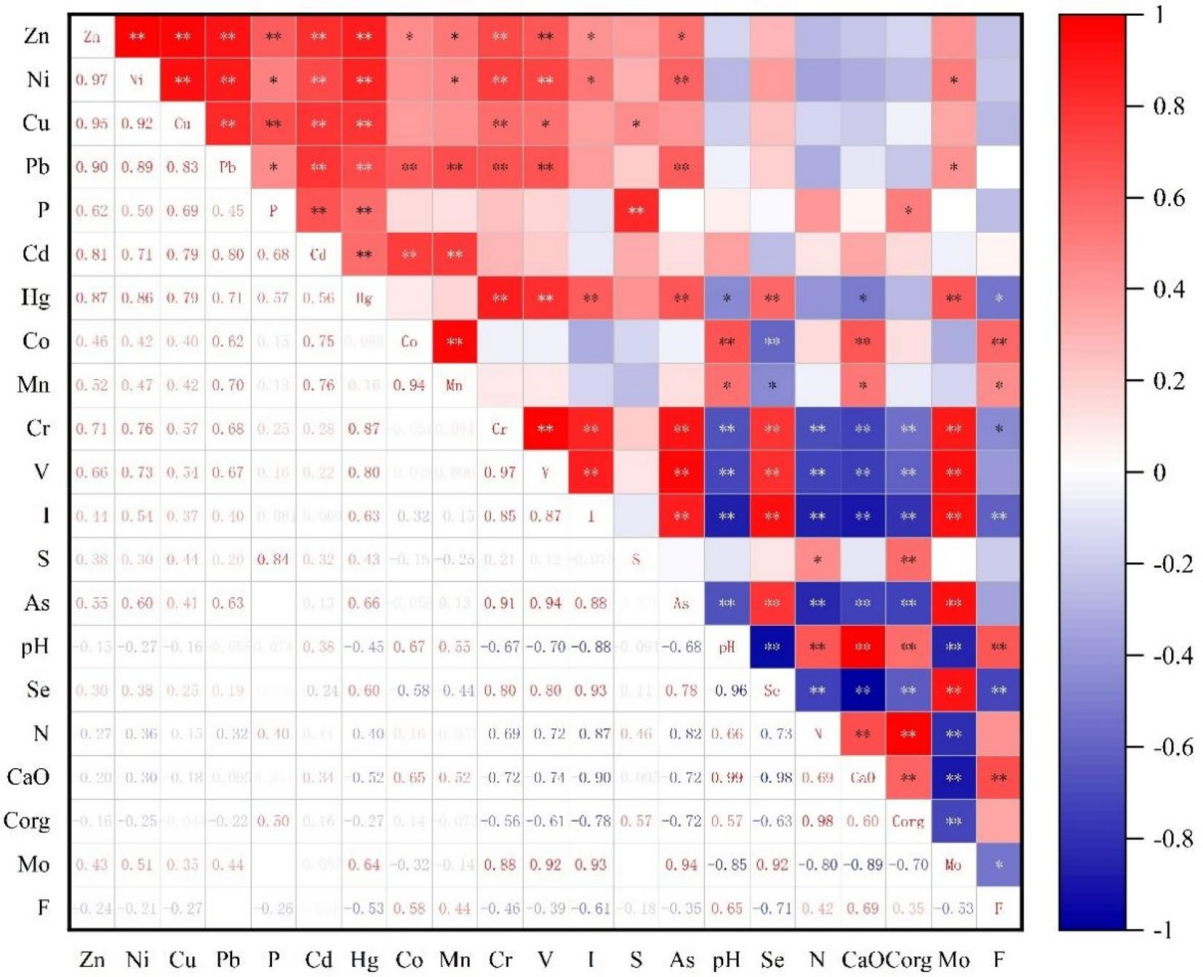
Correlation among 21 elements in the karstic soil.

Through a relevance analysis of the interrelationships among soil elements, we can speculate whether there is homogeneity or source identification among the elements^40^. Figure 5 shows the results of the descriptive statistical analysis of the concentrations of 21 topsoil elements in the Wuming Basin, and the correlation coefficients between different elements were analyzed using Spearman correlation analysis. The results indicated that, except for P and F, Hg and F, S, pH, and Corg and Mo, there were significant correlations (P < 0.01) between all the other elements. Among them, Zn–Ni, Zn–Cu, Zn–Pb, Zn–Cd, Zn–Hg, Zn–Cr, Zn–V, Ni–Cu, Ni–Pb, Ni–V, Pb–Cd, Cr–V, Cr–As, V–As, V–Mo, I–Se, As–Mo, pH-CaO, and N-Corg have correlation coefficients (R^2^) exceeding 0.7, indicating a strong correlation, while the correlations between the remaining elements were moderate to weak. In summary, Zn, Ni, Cu, Pb, Cd, and Hg may share a common origin; As, Cr, V, and Mo may have the same source; I and Se may have the same source; N and C may have the same source; and Ca and pH are probably of the same origin.

#### PCA

Bartlett's sphericity test result (0.00 < 0.05) and the Kaiser–Meyer–Olkin test result (0.833 > 0.5) indicate a strong correlation among the elements (Table S8), making it suitable for conducting PCA. Using Kaiser normalization and the orthogonal rotation method, five common factors were extracted based on the criterion of eigenvalues greater than one, accounting for a cumulative variance contribution of 70.6%. The extracted common factors can effectively explain most of the information on all elements.

The first factor accounted for 31.691% of the total variance, making it the most significant factor, with substantial loadings for Zn, Ni, Cu, Pb, P, Cd, and Hg. Previous studies indicated that Zn, Ni, Cu, Pb were frequently enriched in the weathering zone of basalt, with concentrations much higher in basic rocks compared to other elements ^41–43^. Zn, Ni, Cu, Pb, P, Cd, and Hg are considered typical compatible elements, often associated with the weathering of basic rocks. When examining the factor score map, geological map, and soil type distribution map, the high concentration areas closely aligned with the Devonian melting county formation and limestone formation, especially in the western and southwestern regions where lime soils are prevalent, indicating a geological origin. Research has shown that livestock manure and its processed products (organic fertilizers) contain higher levels of Cu and Zn, contributing significantly to the accumulation of heavy metals in farmland soils^44^. The use of fertilizers and pesticides has been shown to lead to the accumulation of Cu, Pb, Cd, and Hg to some extent^7,45–48^. Zn, Ni, Cu, Pb, Cd, and Hg exhibited a relatively high coefficient of variation, suggesting potential anthropogenic sources. Examining the land use types, the high concentration areas were widely distributed in dryland, forest, and tea plantations. In this region, the accumulation of Zn, Cu, Pb, Cd, and Hg may be associated with the application of fertilizers (both inorganic and organic, particularly manure) and pesticides in dryland, forest, and tea plantations, indicating agricultural activities as a potential source. In conclusion, the elements represented by this factor likely have a combined origin from geological sources and agricultural activities.

The second factor contributed to 15.656% of the total variance, with significant loadings for Se, Mo, V, I, As, and Cr. Qian Jianxun et al.^49^ conducted a study on Se in the topsoil of Wuming County, indicating that the spatial distribution of soil Se is closely related to geological factors such as strata, folds, and rivers, influenced by the geological background. Research by Kun Lin et al.^23^ demonstrated a high enrichment of Mo in the surface soil of karst areas with developed carbonate rocks. Comparing the factor score map with the geological map, high-value areas were mainly distributed in the Permian, limestone, and Devonian Lianhua Formation, Nagao Formation, Yuejiang Formation, and Tangding Formation-Luofu Formation. The lithology of these formations is mainly composed of carbonate rocks and clastic rocks. Combining this with the soil distribution map, some high-value areas overlapped with the distribution of lime soil, puddle-cultivated paddy soil, and submerged-cultivated paddy soil. Lime soil develops from limestone parent material, and limestone is a type of carbonate rock. The parent material of paddy soil consists of up to seven different types^50^. Mo, V, and Cr are considered deep-water sedimentary elements, classified as having a geological origin. As for the potential anthropogenic sources, urban household waste (such as batteries, plastics, discarded tires, and colored glass) contains a considerable amount of As^51^, and Cr pollution sources include electroplating, leather wastewater, and chromium slag. Mo, As, Cr, and I exhibited coefficients of variation exceeding 0.5, indicating potential anthropogenic sources. Considering the land use type map, the high-value areas were widely distributed with rivers, urban areas, and factories. In summary, this factor is mainly a mixed source from both geological background and human activities.

The third factor, with heavily loaded elements and soil parameters N, S, and Corg, accounted for 10.125% of the total variance. This represents the nutrient elements in quaternary soils. Surface vegetation and human activity were the main controlling factors. The CV of N, S, and Corg were all less than 0.5, indicating that these factors had few anthropogenic sources and were mainly of natural origin. Comparing the factor score map with the land use type map (Fig. S2), the high-concentration regions were widely associated with paddy fields and the enrichment coefficients of N, S, and Corg in the paddy fields were relatively high. According to Table S2, the N and Corg contents did not differ much from the soil background values of the Guangxi Beibu Gulf Economic Zone, indicating no external input. Therefore, this factor was mainly derived from surface vegetation.

**Figure 6:**
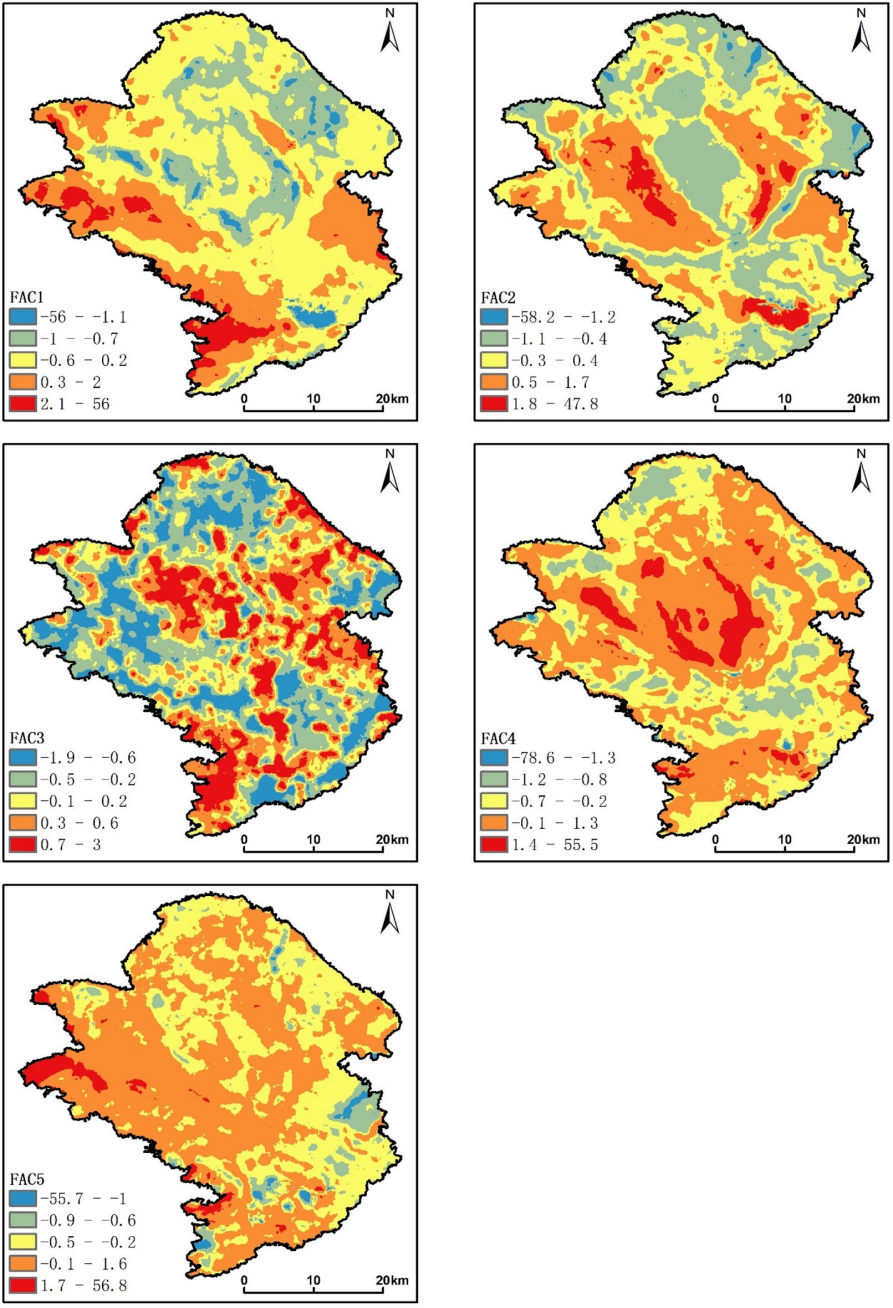
Factor score map. Software: ArcGIS 10.8. https://www.esrichina.hk/en-hk/home (ESRI, US).

The fourth factor, with the heavily loaded elements being Pb, F, Co, and Mn, accounted for 8.039% of the total variance. Comparing the factor score map (Fig. 6) with the geological map (Fig. 2), the Triassic formations were all within high-concentration areas. Triassic formations have a wide distribution of carbonate, clastic, and acidic rocks, and Pb, F, Co, and Mn are highly enriched in the acidic rocks. Lead, cobalt, and manganese showed a large CV, suggesting potential anthropogenic sources. Comparing the factor score map with the land use type map (Fig. S2), high-value areas are widely distributed with industrial and mining enterprises, pipeline transportation, and road land use. There is a large distribution of Mn and Co mines in the study area. Pb can originate from tire wear or incomplete fuel combustion. Therefore, this factor likely represented a mixed source of acidic rocks in the soil parent material, mining activities, and transportation.

The fifth factor contributed to 5.063% of the total variance, with significant loadings for CaO, pH, and Cd. In Guangxi, where carbonate rock areas are widespread, the soil formation process is influenced by the humid and hot climate, leading to substantial leaching of elements such as MgO and K_2_O during soil formation. This results in the secondary enrichment of heavy metals like Cd in the soil, displaying high background characteristics^52^. CaO and Cd exhibited higher enrichment coefficients in acidic rocks, while CaO, pH, and Cd showed higher enrichment coefficients in lime soil. Moreover, the spatial distribution of factor scores coincided with the carbonate rocks of the Devonian and Carboniferous systems in the region, indicating the influence of parent material factors in soil formation. Studies by Bolun Zhang et al.^53^ suggested that the increase in Cd concentration might be related to soil CaO concentration and alkaline soil conditions. Lime is commonly added to improve soil fertility. Correlation analysis indicated a strong relationship between CaO content and soil pH. CaO and Cd showed large coefficients of variation, indicating strong variability, with a significant anthropogenic influence. The high-value areas on the factor score map coincided with the distribution of lime companies, suggesting that CaO is primarily of anthropogenic origin. Cd mainly originates from industrial wastewater discharge. In summary, the elements represented by this factor have a mixed origin from parent material and industrial sources.

### APCS-MLR source analysis

The APCS-MLR method was employed to calculate the origins and contributions of various elements in the soil, and the results are shown in Table 1. Except for P, Hg, and Mo, the adjusted R^2^ values for the remaining elements were all above 0.6, indicating a good fit and high reliability of the APCS-MLR analysis. In the topsoil of the research region, Ni and Zn were mainly influenced by geology, agricultural fertilization, and pesticides, with contributions of 37.99% and 35.07%, respectively. In contrast, Mo, V, Cr, Se, Hg, and As were primarily affected by the geological background and human activities, with contributions of 59.22%, 55.67%, 50.96%, 41.87%, 40.01%, and 39.44%, respectively. Several studies^54–56^ have indicated that the sources of I in the soil are mainly attributed to four categories: parent material, atmosphere, ocean, and plant bodies. Apart from having 31.94% originating from geological background and human activities, I is also influenced by unknown sources, contributing to 31.95%. The enrichment of I in plant bodies is generally much lower than in the soil, and since the region is a non-marine area, I is likely to primarily originate from parent material and the atmosphere. Corg, S, N, and P were mainly derived from surface vegetation, with contributions of 80.33%, 71.22%, 70.03%, and 45.39%, respectively. Conversely, F, Co, Mn, and Pb were mainly sourced from acidic rocks in the soil parent material, mining activities, and transportation, with contributions of 47.93%, 47.41%, 35.24%, and 31.63%, respectively. Cd and CaO were influenced by parent materials and industrial activities, with contributions of 31.67% and 40.23%, respectively. For Cu, Cd, pH, and CaO, unknown sources contributed 30.65%, 38.75%, 58.95%, and 40.85%, respectively, which requires further investigation. The Cu content was found to be relatively high in Devonian and Carboniferous strata distribution areas^57^, whereas Cd was commonly considered to originate from compound fertilizers, pesticides, poultry, and livestock manure, and rural domestic waste^58^.

**Table 1:** Results of APCS-MLR analysis for farmland soil in the study area (%).

元素	F1, %	F2, %	F3, %	F4, %	F5, %	Unkonwn source, %	R^2^	Adjusted R^2^
Zn	35.07	22.21	8.94	7.34	13.20	13.25	0.898	0.898
Ni	37.99	32.91	9.24	7.49	9.75	2.62	0.819	0.818
Cu	26.51	6.91	20.01	5.74	10.17	30.65	0.632	0.632
Pb	20.76	20.45	13.40	31.63	2.66	11.10	0.766	0.766
P	22.10	5.06	45.39	1.98	0.40	25.07	0.585	0.585
Cd	17.44	0.43	2.03	9.68	31.67	38.75	0.71	0.71
Hg	31.63	40.01	5.71	3.63	13.86	5.16	0.445	0.444
Co	16.67	0.99	3.17	47.41	16.49	15.26	0.845	0.845
Mn	15.13	2.25	20.06	35.24	13.48	13.84	0.655	0.655
Cr	18.05	50.96	8.26	5.76	12.65	4.33	0.649	0.649
V	13.62	55.67	10.52	15.81	2.20	2.19	0.702	0.702
I	7.81	31.94	17.45	4.29	6.56	31.95	0.653	0.653
S	8.23	11.22	71.22	4.97	2.81	1.55	0.656	0.655
As	4.24	39.44	3.01	29.59	1.15	22.56	0.614	0.614
pH	1.00	7.13	8.78	8.42	15.72	58.95	0.672	0.672
Se	1.36	41.87	6.85	11.10	7.38	31.44	0.803	0.803
N	1.18	5.35	70.03	5.46	5.99	11.99	0.879	0.879
CaO	0.59	1.75	13.79	2.80	40.23	40.85	0.728	0.728
Corg	1.09	2.08	80.33	3.71	3.86	8.92	0.846	0.846
Mo	1.15	59.22	3.20	11.11	3.96	21.36	0.565	0.565
F	5.91	6.88	25.73	47.93	1.24	12.31	0.7	0.7

“–” represents that the source contribution value is very small and can be negligibly ignored.

In this study, we conducted a comprehensive investigation of PTEs and other elements in the soil of the Wuming Basin. Research on the benchmark values based on different geological units, soil types, and land use types laid the foundation for the formulation of soil environmental quality standards in the region. The assessment of the pollution status and potential ecological risks of several PTEs provides a basis for optimizing the environment in the Wuming Basin. This study elucidates the sources and contributions of 21 elements in the Wuming Basin and provides a research basis for the safe utilization of land and soil remediation in this region. Moreover, it can serve as a scientific basis for future studies on soil elements in other karst areas and for the sustainable utilization and management of soil resources.

## Conclusion

This study proposes the geochemical baseline values for 21 common elements in high geological background areas of karst regions. It quantitatively analyzes the sources of these 21 elements and examines the characteristics of potentially toxic elements (PTEs) pollution in the study area. Unlike previous studies that focused mainly on As and a few other PTEs^5,7,59^, the scope of this research expanded. The results of the baseline values for the 21 elements based on different geological units, soil types, and land use types are presented in Tables S3–S5. Ni and Zn in the soil originate from geological processes, agricultural fertilization, and pesticides, contributing to 37.99% and 35.07%, respectively. Mo, V, Cr, Se, Hg, and As have sources from both geological background and human activities, ranging from 39.44% to 59.22%. Corg, S, N, and P come predominantly from surface vegetation, accounting for 45.39% to 80.33%. F, Co, Mn, and Pb derive from soil parent material, including acidic rocks, mining activities, and transportation, contributing to 31.63% to 47.93%. Cd and CaO originate from both soil formation processes and industrial activities, contributing to 31.67% and 40.23%, respectively. I has sources from geological background and human activities (31.94%) as well as soil formation processes and the atmosphere (31.95%). Cu is primarily sourced from geological processes, accounting for 30.56%. The overall pollution level of Cr, Pb, As, and Zn in the study area is categorized as mild, while Hg poses a moderate ecological risk. The overall ecological risk in the study area is considered mild to moderate. In conclusion, this study establishes a foundation for the formulation of soil environmental quality standards in this region and other karst areas. It provides a scientific basis for future land use, soil remediation, and soil element studies. Although the study collected a large number of samples in the 1380 km^2^ study area, which resulted in accurate results, it incurred high costs. Future research could refine the sampling strategy by identifying an optimal sampling density with fewer points but low cost and effective results. The geochemical characteristics of soil elements in this study are mainly influenced by geological structures and anthropogenic sources. Future research could focus on studying the temporal variations of soil elements.

### Supplementary Information

Supplementary Information.

## Data Availability

Data will be provided upon request to the corresponding author.
