# Supplementary material for: Exposure to arsenic and other potentially toxic elements: health risk assessment and source analysis in the Wuming Basin, Guangxi Province, China
Source: Sci Rep. 2024 Feb 3;14:2835. doi: 10.1038/s41598-024-52947-y (PMC10838325; doi:10.1038/s41598-024-52947-y)
Supplement: Supplementary file 1 — Supplementary Information. [file 41598_2024_52947_MOESM1_ESM.docx]

**Exposure to Arsenic and Other Potentially Toxic Elements: Health Risk Assessment and Source Analysis in the Wuming Basin, Guangxi Province, China**

HU Bo^1,2^ LI Jie^3^  LIU Rui^4,^* LEI Guoxin^4^ WANG Xinyu^3^ WANG Lei^3^

1. Key Laboratory of Environment Change and Resources Use in Beibu Gulf, Ministry of Education,Nanning Normal University, 175 Mingxiu East St., Nanning 530001, China;
2. School of Environmental and Life Sciences，Nanning Normal University, Nanning 530001, China;
3. Geological Survey of Guangxi Zhuang Autonomous Region, Nanning, 530023, P. R. China;
4. College of Resources and Environment, Yangtze University, Wuhan, 430100, China

* E-mail: [Liuxiaoxiao_Rr@163.com](mailto:Liuxiaoxiao_Rr@163.com)


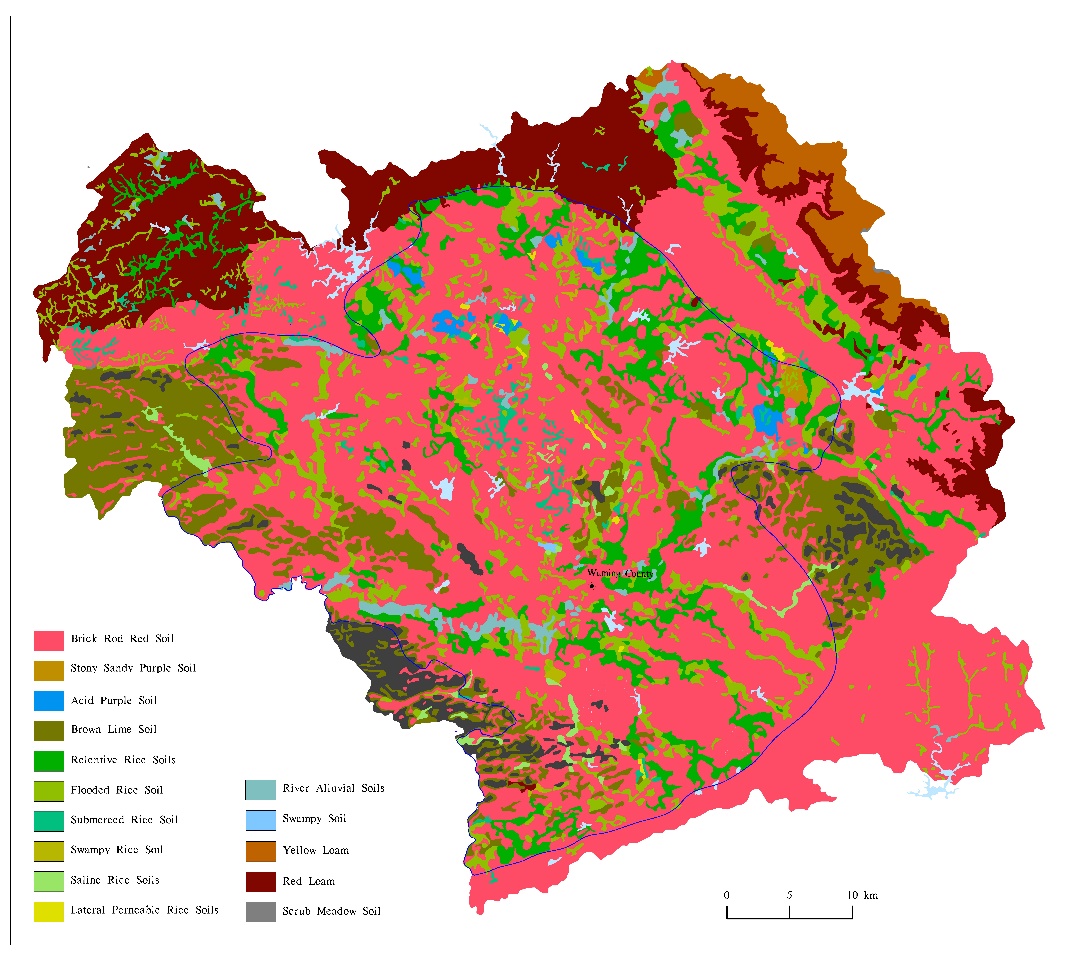


**Fig.S1** Map of Soil Types in the Research Zone. Software: MapGIS 6.7. URL: <https://www.mapgis.com/> (Wuhan Zhongtian Network Co., Ltd.)


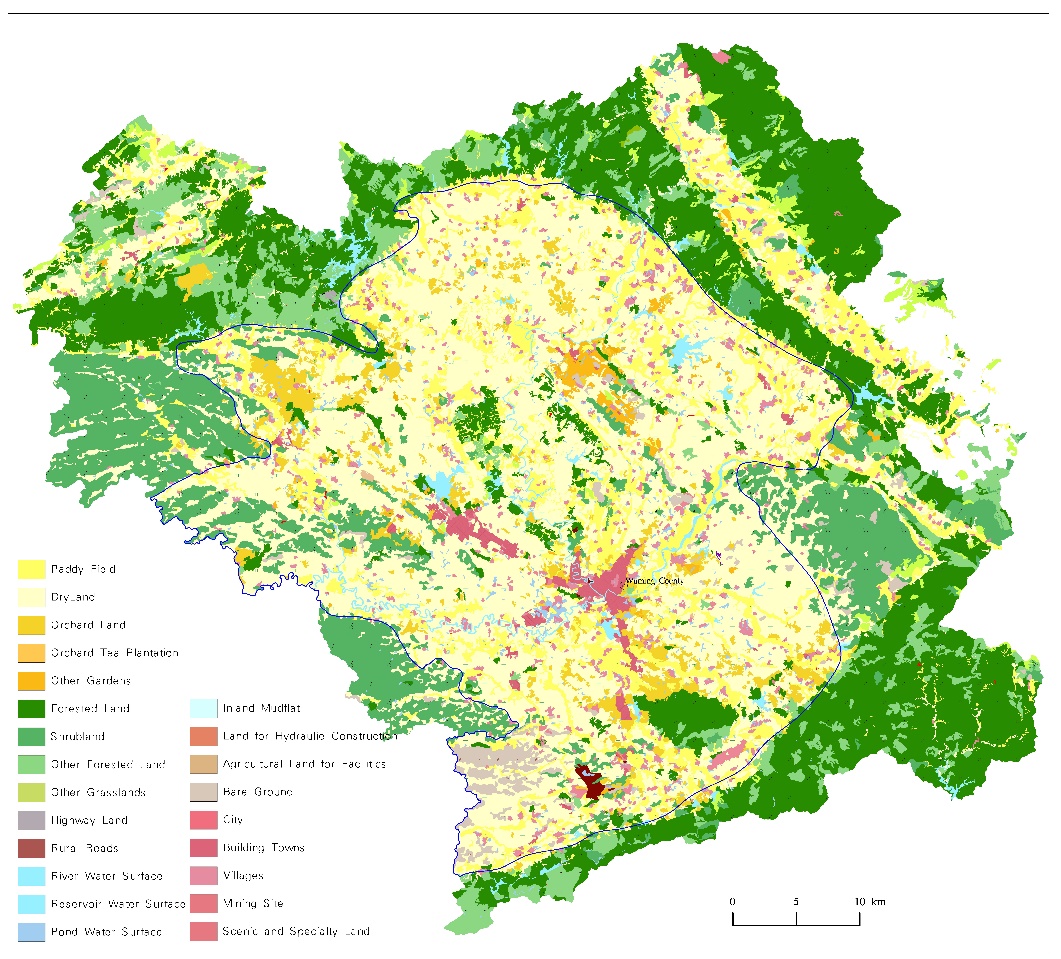


**Fig.S2** Map of Land Use Types in the Research Zone. Software: MapGIS 6.7. URL: <https://www.mapgis.com/> (Wuhan Zhongtian Network Co., Ltd.)

**Table S1** Criteria for Classification of Heavy Metal Contamination of Soil and Evaluation of Ecological Risks

| I_geo_ | Degree of Contamination | E_r_i | Potential Ecological Risk Level | RI | Potential Ecological Risk Level |
| --- | --- | --- | --- | --- | --- |
| I_geo_≤0 | None | E_r_i<40 | Slight | RI<150 | Slight |
| 0＜I_geo_≤1 | Light | 40＜E_r_i≤80 | Moderate | 150＜RI≤300 | Moderate |
| 1＜I_geo_≤2 | Medium | 80＜E_r_i≤160 | Strong | 80＜RI≤160 | Strong |
| 2＜I_geo_≤3 | Medium-Intense | 160＜E_r_i≤320 | Very Strong | 300＜RI≤600 | Very Strong |
| 3＜I_geo_≤4 | Intense | E_r_i>320 | Extremely Strong | RI>600 | Extremely Strong |
| 4＜I_geo_≤5 | Intense-Very Intense |  |  |  |  |
| I_geo_＞5 | Very Intense |  |  |  |  |

**Table S2** Descriptive Statistics

| Elements | In This Research | | | | | | | | Soil Background Value in China | Soil Background Value in Guangxi | Soil Background Value in Beibu Gulf Economic Zone of Guangxi |
| --- | --- | --- | --- | --- | --- | --- | --- | --- | --- | --- | --- |
|  | Number of samples | Skewness | Kurtosis | Maximum | Minimum | Mean | Standard Deviation | Coefficient of Variation |  |  |  |
| Zn | 12547 | 9.33 | 99.53 | 20.40 | 999.70 | 132.07 | 89.42 | 0.68 | 74.00 | 75.60 | 43.38 |
| Ni | 12547 | 5.76 | 49.01 | 5.40 | 209.60 | 37.94 | 23.09 | 0.61 | 27.00 | 26.60 | 14.86 |
| Cu | 12547 | 9.19 | 215.06 | 5.50 | 520.40 | 34.02 | 18.87 | 0.55 | 23.00 | 27.80 | 17.88 |
| Pb | 12547 | 10.60 | 232.66 | 9.40 | 376.40 | 40.98 | 21.36 | 0.52 | 26.00 | 24.00 | 22.35 |
| P | 12547 | 16.31 | 825.51 | 179.00 | 24061.70 | 926.42 | 450.84 | 0.49 | —— | —— | 615.66 |
| Cd | 12547 | 3.31 | 20.81 | 0.03 | 16.19 | 0.52 | 0.89 | 1.71 | 0.10 | 0.27 | 0.14 |
| Hg | 12547 | 1.68 | 4.63 | 8.00 | 8980.00 | 181.98 | 155.72 | 0.86 | 65.00 | 152.00 | 82.88 |
| Co | 12547 | 1.56 | 2.47 | 1.10 | 87.10 | 11.47 | 7.89 | 0.69 | 13.00 | 10.40 | 6.03 |
| Mn | 12547 | 1.63 | 3.77 | 43.30 | 29640.00 | 552.57 | 781.00 | 1.41 | 585.00 | 446.00 | 159.32 |
| Cr | 12547 | 0.30 | 1.01 | 23.30 | 1126.90 | 182.67 | 116.08 | 0.64 | 61.00 | 82.10 | 49.79 |
| V | 12547 | 1.19 | 1.14 | 30.50 | 2666.00 | 176.66 | 83.57 | 0.47 | 82.00 | 129.90 | 70.96 |
| I | 12547 | 5.81 | 94.55 | 0.44 | 24.99 | 7.66 | 4.85 | 0.63 | 3.80 | 7.93 | 2.55 |
| S | 12547 | 1.92 | 12.41 | 72.80 | 3686.20 | 278.69 | 104.93 | 0.38 | —— | —— | —— |
| As | 12547 | 12.14 | 562.21 | 0.40 | 461.80 | 39.62 | 32.73 | 0.83 | 11.00 | 20.50 | 7.96 |
| pH | 12547 | 6.38 | 141.62 | 3.22 | 8.42 | 5.29 | 0.97 | 0.18 | —— | —— | —— |
| Se | 12547 | 3.00 | 17.63 | 0.07 | 9.04 | 0.85 | 0.39 | 0.46 | 0.29 | 0.77 | 0.52 |
| N | 12547 | 1.85 | 21.61 | 302.00 | 4581.00 | 1275.01 | 493.60 | 0.39 | —— | —— | 1185.52 |
| CaO | 12547 | 1.32 | 2.81 | 0.02 | 28.35 | 0.50 | 1.72 | 3.47 | 2.20 | 0.13 | 0.17 |
| Corg | 12547 | 6.42 | 156.15 | 0.20 | 6.08 | 1.30 | 0.48 | 0.37 | —— | —— | 1.29 |
| Mo | 12547 | 1.02 | 2.33 | 0.30 | 70.42 | 2.21 | 2.18 | 0.99 | 2.00 | 5.90 | 0.79 |
| F | 12547 | 0.95 | 0.47 | 145.00 | 3715.00 | 506.88 | 233.99 | 0.46 | 480.00 | 481.00 | 378.24 |
| "— " to indicate that there is no relevant data in the article  pH is dimensionless, CaO and Corg in %, Hg in μg/kg, and the rest of the elements in mg/kg. | | | | | | | | | | | |

**Table S3** Geochemical Baseline Values for Agricultural Soils in Various Geologic Units

| Elements | Fourth century sediments | | acidic rocks | | clastic rocks | | Carbonates Rocks | | Neutral Rocks | |
| --- | --- | --- | --- | --- | --- | --- | --- | --- | --- | --- |
|  | Mean | K | Mean | K | Mean | K | Mean | K | Mean | K |
| CaO | 0.18 | 0.9 | 0.37 | 1.85 | 0.23 | 1.15 | 0.18 | 0.9 | 0.18 | 0.9 |
| Corg | 1.18 | 0.95 | 1.43 | 1.15 | 1.27 | 1.02 | 1.27 | 1.02 | 1.11 | 0.9 |
| As | 26.3 | 0.81 | 49.5 | 1.52 | 14.4 | 0.44 | 45.7 | 1.4 | 17.7 | 0.54 |
| Cd | 0.23 | 0.88 | 0.318 | 1.21 | 0.191 | 0.73 | 0.278 | 1.06 | 0.102 | 0.39 |
| Co | 7.4 | 0.8 | 18.8 | 2.04 | 13.8 | 1.5 | 10.1 | 1.1 | 7.9 | 0.86 |
| Cr | 136.7 | 0.88 | 149.8 | 0.97 | 91.4 | 0.59 | 200.85 | 1.3 | 82 | 0.53 |
| Cu | 29.9 | 0.99 | 23.6 | 0.78 | 34.55 | 1.14 | 31.5 | 1.04 | 20.9 | 0.69 |
| F | 404 | 0.9 | 816 | 1.81 | 517 | 1.15 | 455 | 1.01 | 461 | 1.02 |
| Hg | 149 | 1.03 | 99 | 0.68 | 80 | 0.55 | 173 | 1.19 | 60 | 0.41 |
| I | 6.775 | 0.89 | 4.615 | 0.6 | 5.085 | 0.67 | 9.76 | 1.28 | 4.365 | 0.57 |
| Mn | 218.6 | 0.71 | 833.45 | 2.71 | 360.1 | 1.17 | 328.15 | 1.07 | 198.2 | 0.64 |
| Mo | 1.59 | 0.87 | 1.95 | 1.07 | 0.84 | 0.46 | 2.36 | 1.3 | 0.83 | 0.46 |
| N | 1129 | 0.96 | 1378 | 1.17 | 1284 | 1.09 | 1208 | 1.02 | 1000 | 0.85 |
| Ni | 28.8 | 0.96 | 24.15 | 0.81 | 30.5 | 1.02 | 37.1 | 1.24 | 19.75 | 0.66 |
| P | 870.6 | 1.02 | 837.6 | 0.98 | 663.3 | 0.78 | 895 | 1.05 | 601.1 | 0.71 |
| Pb | 31.9 | 0.93 | 56.9 | 1.65 | 28.8 | 0.84 | 43.4 | 1.26 | 23.15 | 0.67 |
| S | 267.95 | 1.01 | 267.5 | 1 | 234.45 | 0.88 | 277.1 | 1.04 | 204.3 | 0.77 |
| Se | 0.782 | 1.01 | 0.584 | 0.76 | 0.575 | 0.74 | 0.913 | 1.18 | 0.494 | 0.64 |
| V | 157.8 | 0.94 | 197.9 | 1.81 | 137.4 | 0.82 | 203 | 1.21 | 97.3 | 0.58 |
| Zn | 94.8 | 0.98 | 90.9 | 0.94 | 84.2 | 0.87 | 120.1 | 1.24 | 60.8 | 0.63 |

K represents the enrichment factor, K = statistical unit background value/study area background value

**Table S4** Geochemical Baseline Values for Chemical Elements in Different Types of Soils of Study Area

| Elements | Red Soil | | Lime Soil | | Rice Soil | | Alluvial Soil | | Purple Soil | |
| --- | --- | --- | --- | --- | --- | --- | --- | --- | --- | --- |
|  | Mean | K | Mean | K | Mean | K | Mean | K | Mean | K |
| CaO | 0.16 | 0.8 | 0.25 | 0.589 | 0.22 | 1.1 | 0.225 | 1.13 | 0.23 | 1.15 |
| Corg | 1.23 | 0.99 | 1.33 | 1.07 | 1.22 | 0.98 | 0.99 | 0.8 | 1.12 | 0.9 |
| pH | 4.91 | 0.97 | 5.415 | 1.07 | 5.23 | 1.03 | 5.23 | 1.03 | 5.18 | 1.02 |
| As | 33.9 | 1.04 | 50.8 | 1.56 | 22.8 | 0.7 | 20.6 | 0.63 | 13.5 | 0.41 |
| Cd | 0.211 | 0.81 | 0.794 | 3.03 | 0.227 | 0.87 | 0.216 | 0.82 | 0.153 | 0.58 |
| Co | 8.6 | 0.93 | 18.15 | 1.97 | 8.6 | 0.93 | 6.9 | 0.75 | 11.5 | 1.25 |
| Cr | 161.9 | 1.05 | 243.1 | 1.57 | 119.55 | 0.77 | 96.4 | 0.62 | 91 | 0.59 |
| Cu | 30.4 | 1 | 49 | 1.62 | 27.7 | 0.91 | 23.7 | 0.78 | 25.2 | 0.83 |
| F | 433 | 0.96 | 466 | 1.03 | 450 | 1 | 400 | 0.89 | 507 | 0.12 |
| Hg | 142 | 0.98 | 312 | 2.15 | 124 | 0.85 | 113 | 0.78 | 61 | 0.42 |
| I | 8.95 | 1.17 | 10.78 | 1.41 | 3.965 | 0.52 | 3.71 | 0.49 | 3.74 | 0.49 |
| Mn | 261.1 | 0.85 | 811.6 | 2.64 | 251.8 | 0.82 | 192.1 | 0.62 | 319 | 1.04 |
| Mo | 1.9 | 1.04 | 2.35 | 1.29 | 1.44 | 0.79 | 1.07 | 0.59 | 0.68 | 0.37 |
| N | 1152 | 0.98 | 1300.5 | 1.1 | 1179 | 1 | 944 | 0.8 | 1086 | 0.92 |
| Ni | 31.3 | 1.04 | 68.5 | 2.28 | 24.9 | 0.83 | 21.2 | 0.71 | 26.6 | 0.89 |
| P | 826.8 | 0.97 | 1137.95 | 1.34 | 835.65 | 0.98 | 765.8 | 0.9 | 602.4 | 0.71 |
| Pb | 34.7 | 1.01 | 67 | 1.95 | 30.9 | 0.9 | 26.8 | 0.78 | 26.55 | 0.77 |
| S | 267.2 | 1 | 309.45 | 1.16 | 256.35 | 0.96 | 211.2 | 0.79 | 203.35 | 0.76 |
| Se | 0.864 | 1.12 | 0.81 | 1.05 | 0.641 | 0.83 | 0.611 | 0.79 | 0.437 | 0.57 |
| V | 179.2 | 1.07 | 223.3 | 1.33 | 141.5 | 0.85 | 103.45 | 0.62 | 108.65 | 0.65 |
| Zn | 96.3 | 1 | 265.95 | 2.76 | 84.5 | 0.88 | 80.8 | 0.84 | 72.55 | 0.75 |

**Table S5** Geochemical Baseline Values for Chemical Elements in Different Land Use of Soils of Study

| Elements | Dryland | | Paddy Land | |
| --- | --- | --- | --- | --- |
|  | Mean | K | Mean | K |
| CaO | 0.15 | 0.75 | 0.26 | 1.3 |
| Corg | 1.15 | 0.93 | 1.41 | 1.14 |
| pH | 4.78 | 0.94 | 5.6 | 1.1 |
| As | 37.1 | 1.14 | 12.6 | 0.39 |
| Cd | 0.204 | 0.78 | 0.188 | 0.72 |
| Co | 8.7 | 0.95 | 7.7 | 0.84 |
| Cr | 174.4 | 1.13 | 83.3 | 0.54 |
| Cu | 30.6 | 1.01 | 25.5 | 0.84 |
| F | 425 | 0.94 | 456.5 | 1.01 |
| Hg | 147.5 | 1.02 | 101 | 0.7 |
| I | 9.85 | 1.29 | 1.69 | 0.22 |
| Mn | 272.95 | 0.89 | 190.35 | 0.62 |
| Mo | 2.06 | 1.13 | 0.65 | 0.36 |
| N | 1092 | 0.93 | 1642 | 1.39 |
| Ni | 33.9 | 1.13 | 20.6 | 0.69 |
| P | 822.2 | 0.97 | 802.7 | 0.94 |
| Pb | 36.4 | 1.06 | 25.2 | 0.73 |
| S | 254.6 | 0.95 | 291.6 | 1.09 |
| Se | 0.923 | 1.2 | 0.506 | 0.66 |
| V | 190.6 | 1.14 | 114 | 0.68 |
| Zn | 103.7 | 1.07 | 76.2 | 0.79 |

**Table S6** Percentage of Class Distribution for Pollution Assessment of Heavy Metals in Study Aera Using Geoacumulation Index

| elements | Min | Max | Mean | Proportion of Sample Points（%） | | | | | | |
| --- | --- | --- | --- | --- | --- | --- | --- | --- | --- | --- |
|  |  |  |  | ≤0 | 0-1 | 1-2 | 2-3 | 3-4 | 4-5 | ≥5 |
|  |  |  |  | None | Light | Medium | Medium-  Intense | Intense | Intense-  Very  Intense | Very  Intense |
| Zn | -2.40 | 3.22 | 0.02 | 57.0 | 26.06 | 16.22 | 0.69 | 0.01 | 0.00 | 0.00 |
| Ni | -2.82 | 2.46 | -0.25 | 63.93 | 27.32 | 8.70 | 0.05 | 0.00 | 0.00 | 0.00 |
| Cu | -2.86 | 3.71 | -0.38 | 73.51 | 24.88 | 1.45 | 0.11 | 0.05 | 0.00 | 0.00 |
| Pb | -1.89 | 3.44 | 0.07 | 50.58 | 37.90 | 11.39 | 0.11 | 0.02 | 0.00 | 0.00 |
| Cd | -4.41 | 4.62 | -1.17 | 81.80 | 10.50 | 4.61 | 2.35 | 0.68 | 0.06 | 0.00 |
| Hg | -4.76 | 5.37 | -0.58 | 71.91 | 22.84 | 4.94 | 0.29 | 0.02 | 0.00 | 0.01 |
| Cr | -2.33 | 3.27 | 0.39 | 35.20 | 40.69 | 21.05 | 3.04 | 0.02 | 0.00 | 0.00 |
| As | -6.20 | 3.98 | 0.06 | 45.25 | 37.20 | 14.78 | 2.44 | 0.34 | 0.00 | 0.00 |

**Table S7** Potential Ecological Risk Coefficient for Every Heavy Metal in Soils

| elements | Min | Max | Mean | Proportion of Sample Points（%） | | | | |
| --- | --- | --- | --- | --- | --- | --- | --- | --- |
|  |  |  |  | ＜40 | 40-80 | 80-160 | 160-320 | ＞320 |
|  |  |  |  | Slight | Moderate | Strong | Very Strong | Extremely  Strong |
| Zn | 0.28 | 13.94 | 1.84 | 100.00 | 0.00 | 0.00 | 0.00 | 0.00 |
| Ni | 1.06 | 41.21 | 7.46 | 99.99 | 0.01 | 0.00 | 0.00 | 0.00 |
| Cu | 1.03 | 97.89 | 6.40 | 99.90 | 0.08 | 0.02 | 0.00 | 0.00 |
| Pb | 2.03 | 81.26 | 8.85 | 99.92 | 0.07 | 0.01 | 0.00 | 0.00 |
| Cd | 2.11 | 1102.75 | 35.69 | 78.95 | 12.21 | 5.14 | 2.71 | 0.99 |
| Hg | 2.21 | 2484.09 | 50.34 | 50.16 | 34.10 | 14.30 | 1.35 | 0.08 |
| Cr | 0.60 | 28.87 | 4.68 | 100.00 | 0.00 | 0.00 | 0.00 | 0.00 |
| As | 0.20 | 236.21 | 20.26 | 92.22 | 6.44 | 1.25 | 0.09 | 0.00 |

**Table S8** KMO and Bartlett's Test

| KMO and Bartlett's Test | |
| --- | --- |
| KMO Sample Suitability Quantity | 0.833 |
| Bartlett's Test of Sphericity | Approximate Chi-square 181327.7 |
|  | Degrees of Freedom 210 |
|  | Significance 0 |
